# Fear of COVID-19 Impact on Professional Quality of Life among Mental Health Workers

**DOI:** 10.3390/ijerph19169949

**Published:** 2022-08-12

**Authors:** Pentagiotissa Stefanatou, Lida-Alkisti Xenaki, Ioannis Karagiorgas, Angeliki-Aikaterini Ntigrintaki, Eleni Giannouli, Ioannis A. Malogiannis, George Konstantakopoulos

**Affiliations:** First Department of Psychiatry, Eginition Hospital, School of Medicine, National and Kapodistrian University of Athens, 11528 Athens, Greece

**Keywords:** fear of COVID-19, mental health workers, compassion satisfaction, compassion fatigue, burnout, secondary traumatic stress, ProQOL, Greece

## Abstract

Several studies have examined the impact of the COVID-19 pandemic on healthcare workers’ mental health, but only a few have investigated its detrimental effect on the mental well-being of mental health workers (MHWs). Background: The current study aimed to explore the effect of the fear of COVID-19 (FCV-19) on professional quality of life dimensions, namely compassion satisfaction (CS), burnout (BO), and secondary traumatic stress (STS) in MHWs above and beyond sociodemographic and professional factors. Methods: Hierarchical linear regression models were employed to examine the relationship of extreme FCV-19 with CS, BO, and STS in MHWs (*n* = 224), after considering sociodemographic variables as potential confounding factors. Extreme FCV-19 was operationalized as a binary variable with a cut-off score of ≥16.5 considered as extreme fear. Results: We found that extreme FCV-19 in MHWs is linked with increased compassion fatigue (BO and STS), and this relationship is exacerbated by younger age in regard to BO and by female gender concerning STS. CS remains unaffected by severe FCV-19, and it is higher in older participants. Conclusion: Organizational support is required to protect MHWs’ mental well-being and ensure the quality of care they provide during prolonged crises, such as the COVID-19 pandemic. Measures that intensify a sense of safety, protection, and control against COVID-19 infections in mental health services should be included in the recommendations that may reduce BO and STS among MHWs.

## 1. Introduction

The ongoing pandemic of the coronavirus disease 2019 (COVID-19) has been recognized as a substantial global threat [1] that has been widely linked with devastating physical and life-threatening implications for the general population [2]. Concurrently, it has elicited a multilevel psychological impact characterized largely by fear [3], which is precipitated by the knowledge of the virus’s mode of contraction and rates of transmission, morbidity, and mortality [4]. The immense medical needs that emerged placed a heavy burden on the shoulders of the health care providers. Healthcare workers’ (HCWs) mental health has been put forward as a matter of serious consideration during the current pandemic [5]. The increased risk of contracting COVID-19 due to close encounters with the infected patients, fears of transmitting the infection to family and colleagues, and fears of dying due to the increased COVID mortality rate among healthcare workers (HCWs) [6,7] have had substantial psychological consequences for the medical personnel. 

Fear of COVID-19 (FCV-19) has been reported to escalate faster than the virus itself, being greatly related to the uncertainties regarding the outbreak and to negatively affecting the mental state of HCWs and impeding their job performance [8,9]. A recent meta-review addressing the overall mental health of HCWs during the COVID-19 pandemic, identified anxiety, depression, post-traumatic stress disorder, burnout, insomnia, fear, obsessive-compulsive disorder, somatization, phobia, substance abuse, and suicidal thoughts as the most prevailing problems, especially among females and younger persons [10]. In addition, the increasing perceived FCV-19 was robustly associated with a higher prevalence of burnout syndrome and poor quality of life among HCWs [11]. 

As would be expected, the psychological consequences of the pandemic on frontline workers, as those working in acute and emergency settings as well as intensive care units, drew the focus of attention [12]. Τhe psychological wellbeing of the mental health workers (MHWs), both in hospital and in community services, only lately received some attention, despite their crucial role in mitigating the psychiatric effects of the pandemic [13]. A wide range of psychological difficulties due to the COVID-19 pandemic have been observed in MHWs. Because of the aggravation of the already existing mental disorders and the increase in the referrals, MHWs were challenged with the burden of a dramatic change in their working environment [14,15]. The growing professional demands, combined with FCV-19, moral distress due to under-provision of care, and the vicarious traumatization have been linked to feelings of loneliness, irritability, distress, burnout, and maladaptive coping strategies [14,16,17,18]. This holds true especially in high-incidence regions of COVID-19 cases [19]. MHWs providing care for quarantined suspected COVID-19 cases have also reported higher levels of depression and anxiety [20]. In addition, the effect of changes in the professional life of MHWs have been explored qualitatively by Liberati et al. [14] in terms of pausing of services considered to be “non-essential”, modification of in-person services to be performed remotely (e.g., by phone and video-calls), redeployment of staff across frontline mental health settings to unfamiliar roles. These disruptive organizational adaptations in response to the pandemic have been found to impair the quality of MHWs’ working lives [14,21]. 

Burnout, in terms of emotional exhaustion and depersonalization due to prolonged stress has been depicted as the most powerful determinant of psychological problems in MHWs exposed to COVID-19-related workplace stressors [22]. MHWs have also exhibited high resilience, which is an important attribute for coping emotionally with frequent exposure to mortality and psychological distress, especially when accompanied with satisfaction with the organizational support offered [17,23].

Most of the studies that have examined the effects of the COVID-19 pandemic on the mental health of MHWs have concisely concluded that the levels of psychological distress, burnout, post-traumatic stress symptoms, depression, and sleep problems have risen significantly [14,16,17,18,22,24,25] and that systematic support is needed to minimize the complex challenges and to safeguard the mental wellbeing of MHWs. However, only a small number of studies have explored the impact of FCV-19 on MHWs’ mental health [17,22,24,25]. In these studies, FCV-19 was explored, among other work-related risk factors, to merely test its relationship with mental health symptoms and burnout in MHWs. 

To our knowledge, no study so far has explicitly studied the impact of FCV-19 on the job-related quality of life of MHWs independently and beyond other sociodemographic or work-related factors, which can be reflected through the professional quality-of-life (ProQOL) scale [26]. This psychometric tool is divided in two domains reflecting the positive and negative aspects of a person’s carrying out his/her occupation: compassion satisfaction (CS), which constitutes a person’s ability to derive pleasure at work through collegiate collaboration, creative contribution, and provision of help for people at need in the work field and compassion fatigue (CF). The latter is further categorized into the burnout (BO) scale, representing feelings of exhaustion, frustration, anger, and depression, and the secondary trauma stress (STS) scale, representing feelings driven by fear and work-related vicarious trauma. In this study, we examined specific relationships between FCV-19 and ProQOL in MHWs from many different settings. We hypothesized that the fear related to the current pandemic crisis in MHWs’ personal and professional lives, along with the additional emotional labour associated with their work under the present conditions, would affect the MHWs’ efficacy, judgment, and resilience. Thus, the primary research question was whether the extreme fear of COVID-19 in MHWs is related to compassion satisfaction and to dimensions of compassion fatigue, specifically burnout and secondary traumatic stress. We also examined whether these relationships are affected by sociodemographic and occupational factors. 

## 2. Materials and Methods

### 2.1. Design and Participants

This cross-sectional study was conducted between April and June 2021 on 224 MHWs amid the third wave of COVID-19 in Greece [27]. Participant recruitment was conducted by online posts through the professional associations of the various mental health specialties (i.e., psychiatrists, psychologists, occupational therapists, nurses, social workers, counsellors) in Greece. Participants were working in child and adult community mental health centres, psychiatric residential units, day hospitals and day centres, psychiatric hospitals, and psychiatric departments of general hospitals. The inclusion criteria were the following: (a) aged 20–67 years, (b) licensed health professionals, (c) working in community and hospital mental health settings, (d) providing mental health services only in-person rather than remotely (online or by phone) during the period of investigation. 

### 2.2. Assessment Tools

The online survey included questions regarding sociodemographic (age, gender, family status, residence, educational level, marital status, and monthly income) and professional factors (MHWs’ specialties, service setting, professional experience in years), along with the appraisal of the FCV-19 and the ProQOL. Time of completion of the survey was approximately 15–20 min.

Assessment of the severity of the FCV-19 was operationalized through the self-reported fear of COVID-19 scale (FCV-19S). This is a newly reported psychometric instrument originally validated in the Persian language by Ahorsu et al. [4]. The scale has been translated and validated in many other languages so far, including Greek [28]. The scale’s advantage is its brief and comprehensible structure, making its administration simple for clinical and research objectives [29]. It is a seven-item questionnaire (e.g., “I am most afraid of the coronavirus”), rated using a five-point Likert type scale (1 = “strongly disagree”, 2 = “disagree”, 3 = “neither agree nor disagree”, 4 = “agree”, and “5 = strongly agree”). The scores obtained from each item are summed to get the total score (range: 7–35). Higher scores indicate a greater fear of COVID-19. A cut-off point score of 16.5 or higher has been reported to signify significant predictive power for anxiety, health anxiety, and post-traumatic stress symptoms [3].

The quality of life in the working environment was explored through the adaptation of the Greek version of the professional quality of life (ProQOL) [30,31]. This is a well-established self-report questionnaire, which along with its previous versions, has been used in scientific projects in more than 30 countries globally [26]. It consists of 30 items scored on a five-point Likert scale (1 = “never”, 2 = “rarely”, 3 = “sometimes”, 4 = “often”, 5 = “very often”) with higher scores indicating higher levels on each subscale. The three discrete scales, CS, BO, and STS, do not yield a composite score; that is, each subscale is psychometrically unique consisting of 10 items and cannot be combined with the other scores [26].

### 2.3. Data Analysis

The responses of all participants who matched the inclusion criteria were included in the analysis. Extreme FCV-19 was operationalized as a binary variable with a cut-off point score of ≥16.5 considered as extreme FCV-19. CS, BO, and STS were operationalized as continuous variables. The sociodemographic and professional variables that were used included age, gender, educational level, marital status, professional specialty, monthly income, and years of previous experience. The Kolmogorov Smirnov test was employed to examine the normality assumption within each subgroup of FCV-19S (those with extreme FCV-19 and those with not).

We built hierarchical linear regression models to account for the relation of extreme FCV-19S in MHWs with (a) CS, (b) BO, and (c) STS after adjusting for sociodemographic variables as potential confounding factors. We employed all socio-demographic/professional characteristics as block 1 variables and the binary variable reflecting the presence or not of extreme FCV-19 as block 2 variables. Three separate analyses were conducted with each subscale of the ProQOL scale as the dependent variable. *p*-values less than 0.05 were considered significant. All statistical analyses were carried out using IBM SPSS version 24.0 (IBM Corp., Armonk, NY, USA).

## 3. Results

Our sample consisted of 224 MHWs. Descriptive data (age, gender, education level, marital status, job/profession, monthly salary, years of previous experience, FCV-19, CS, BO, and STS) are presented in Table 1. The three sub-scales of ProQOL did not follow the normal distribution in the two subgroups. Hence, preliminary analyses were performed using the Mann–Whitney U test to examine possible differences in the three sub-scales between the two subgroups of FCV-19 (extreme and non-extreme FCV-19). Our findings indicated that MHWs with extreme FCV-19 had statistically higher scores in STS and BO but not statistically different scores in CS (see Table 2).

Table 3 displays the hierarchical regression models predicting ProQOL dimensions. Regarding CS, the first step has an R-square value of 0.081, which can be interpreted as all sociodemographic and professional variables account for 8.1% of the variance in CS scores. When FCV-19 was added in the second step, R-square increased to 0.087 (8.7% of the variance in CS was accounted by all variables in the model). Thus, the addition of extreme FCV-19 explained only 0.6% additional variance in CS, above and beyond what was accounted for by sociodemographic variables. Moreover, the second step was not statistically significant (*p* > 0.05), meaning that the inclusion of FCV-19 produces a non-statistically significant increase in variance accounted for in CS. Only age was a significant predictor in the final model. Specifically, being older by one year increases the average score of CS by 0.183 units.

Regarding BO, the first step has an R-square value of 0.073, which can be interpreted as all sociodemographic and professional variables account for 7.3% of the variance in BO scores. When FCV-19 was added in the second step, R-square increased to 0.124 (12.4% of the variance in BO accounted for by all variables in the model). The addition of extreme FCV-19 contributes to a 5.1% additional variance in the BO score accounted for above and beyond what was accounted for by sociodemographic and professional variables. The second step was statistically significant (*p* < 0.05), meaning that inclusion of the FCV-19 variable produces a statistically significant increase in variance accounted for in the BO score. Age and FCV-19 were the only statistically significant predictors in the final model. More specifically, people with extreme FCV-19 scores on average 3.2 units higher in the BO scale than people with non-extreme FCV-19 and being younger by one year increases the average score of BO score by 0.19 units. 

Regarding STS, the first step has an R-square value of 0.073, which can be interpreted as all sociodemographic and professional variables account for 7.3% of the variance in STS scores. When FCV-19 was added in the second step, R-square increased to 0.135 (13.5% of the variance in STS accounted for by all variables in the model). This can be interpreted as the addition of extreme FCV-19 contributes to a 6.2% additional variance in STS score accounted for above and beyond what was accounted for by sociodemographic and professional variables. The second step was statistically significant (*p* < 0.05), meaning that inclusion of FCV-19 produces a statistically significant increase in variance accounted for in STS score. Only gender and FCV-19 were found to be statistically significant predictors in the final model. More specifically, people with extreme FCV-19 score on average 3.57 units higher in the STS scale than people with non-extreme FCV-19 and being female increases the average score of STS score by 2.7 units.

## 4. Discussion

The present study aimed to explore the impact of fear of COVID-19 on the professional quality of life in MHWs above and beyond the effect of sociodemographic and professional factors. To our knowledge, this is the first study that estimated the independent contribution of FCV-19 to dimensions of ProQOL in MHWs, namely compassion satisfaction, burnout, and secondary traumatic stress. Additionally, no other study has examined the above relationships in MHWs using validated instruments to measure FCV-19 and ProQOL.

As could be expected, our results revealed a lower overall mean FCV-19 score in MHWs (13.76) compared to those reported by HCWs (19.59 to 20.86) both frontline and second line [11,32]. A first explanation of this may be that many HCWs perform their duties in closer proximity to patients than MHWs, which increases the risk of exposure, leading to higher levels of FCV-19 and anxiety. Another potential explanation may be that our study was initiated during the third wave of the pandemic, whereas the aforementioned studies in HCWs were conducted during the outbreak of the coronavirus disease. Some features of the third wave could have been linked to a decreasing sense of danger [33] and, therefore, to the decreased FCV-19 in our study participants: (a) decreasing rates of severe cases and deaths in the general population and among HCWs compared to previous waves, (b) speedy antigen tests which helped in rapid diagnosis and clinical monitoring, (c) the positive results of vaccination against COVID-19 among patients, HCWs [34], and the elderly.T One hundred percent of the study participants have been vaccinated against coronavirus as mandated by the Greek government for all health care personnel. Despite decreased COVID-19 severity and fatality rates during the third pandemic wave, 23.7% of MHWs demonstrated extreme FCV-19. However, this was almost half the proportion of the Greek general population during the first wave (40.3%) [3].

The mean levels of CS and BO reported by MHWs were moderate, whereas they demonstrated low levels of STS. However, participants who exhibited extreme FCV-19 (23.7%) reported moderate levels of STS. According to a recent meta-review [10], our results conform to the rates of BO reported by HCWs worldwide during the third wave [35]. Additionally, these results converge to those of reports indicating low to moderate levels of STS in HCWs during the pandemic [32,36,37]. We found only two studies that examined ProQOL and its dimensions (CS, BO, STS) in MHWs during COVID-19 [38,39]. The moderate mean levels of CS and BO in our study are in accord with those reported by Franza et al. [38] and Minò et al. [39]. However, in these studies, MHWs exhibited greater mean STS levels than our participants with nurses reporting the greatest rate of severe STS. Since the rates of PTSD and STS was found to be greater among nurses than other HCWs by a recent meta-review [10], the lower mean STS level in our study may be due to the reduced proportion of nurses in our sample compared to these prior reports.

We found that extreme fear of COVID-19 in mental health workers is linked with increased compassion fatigue (burnout and secondary traumatic stress), and this relationship is exacerbated by younger age in regard to burnout and by female gender concerning secondary traumatic stress. At the same time compassion satisfaction, which expresses the positive sense of a person’s everyday working experience, seems to remain unaffected by the presence or absence of extreme FCV-19, while it appears to be higher among older participants.

Our results are generally in line with the majority of studies which demonstrated a significant negative impact of FCV-19 on mental health outcomes, work outcomes, quality of life, and job performance in HCWs [8,11,32,40,41,42,43]. However, the comparison of our results with those of the above studies should be considered with caution given that in many of them, FCV-19 was measured through ad hoc questions and not with validated tools. Only a few studies have examined, among other variables, the association of FCV-19 with mental health, well-being, perception of the working environment, and levels of BO in MHWs [17,21,22,24,25]. Specifically, the link between greater BO and severe FCV-19 in our study is similar with the studies by Pappa et al. [17] and Rapisarda et al. [22,24], which reported that worries about self-infection or transferring the illness were positively associated with specific aspects of BO. Additionally, our study demonstrated the FCV-19 impact on BO severity, independently and beyond other variables. BO has been shown as a powerful predictor of unfavourable mental health outcomes in MHWs exposed to COVID-19-related job stressors [22]. Moreover, given the links between BO and decreased productivity, higher clinician turnover, absenteeism, clinician mistakes and accidents, extreme levels of BO may significantly compromise treatment quality, resulting in failure to address patients’ critical clinical and psychological needs [44]. However, the proportion of BO variance explained by FCV-19 in our study was rather modest. One possible explanation for this is that BO among MHWs had already reached alarming levels prior to the epidemic, as previously noted in the literature [44,45]. In Greece, over the past ten years, the substantial underfunding of mental health services and staff shortages due to the prolonged financial crisis [46] led to MHWs performing their tasks under great strain with increased workloads, limited organizational support, and working conditions directly related to increased BO [10,22,47]. Additionally, MHWs are routinely subjected to stressful conditions, especially those caring for patients with serious mental illnesses (SMI), as was the case in our study. Specifically, SMI patients are more susceptible to anxiety symptoms and psychological distress [25]. Furthermore, SMI patients typically present deficits in cognition [48,49], social cognition [50], and psychosocial functioning [51]. Consequently, SMI patients have a greater compliance difficulty with the essential restrictive measures to limit the epidemic, making their daily care by MHWs even more onerous [52]. Furthermore, BO in MHWs may be related to other factors that were not examined in the current report, such as increased workloads [10], decreased social support [47], and lack of adequate protective equipment and facilities [21]. Thus, during the pandemic, these highly demanding working conditions exacerbate pre-existing BO, which is further aggravated by FCV-19. The association between BO and younger age has been previously reported. Specifically, in recent reviews, younger age of HCWs has been indicated as one of the most significant risk factors for BO during the COVID-19 pandemic [10,47]. Younger age may be related to less experience and lack of specialized training, which consequently leads to limited capacity of managing crises or stressful professional situations [47].

Greater levels of STS among MHWs with severe FCV-19 were found in our study. This is an important finding given that STS has been linked to unfavourable mental health outcomes in MHWs and HCWs such as anxiety, depression, and suicidal ideation prior and after the onset of the coronavirus pandemic [16,53,54]. Moreover, studies during the pandemic have also found higher levels of STS in MHWs who reported being more fatigued, doubting their professional competency, as well as those who felt they had a poorer therapeutic relationship with their patients. These adverse STS-related experiences had obvious negative consequences on treatment effectiveness [16]. Furthermore, a positive association between STS and severe FCV-19 has also been found in prior reports examining determinants of STS [55] or PTSD [43] in HCWs. However, in the only study in MHWs that examined FCV-19 as a predictor of PTSD, among other factors, no significant relationship emerged [25]. STS and PTSD are highly overlapping syndromes since STS in MHWs is characterized by symptoms of reliving, avoidance, and hyperarousal in response to the traumatized patients with whom they are empathically engaged [30]. According to our findings, traumatic impact when dealing with secondary exposure to stressful events is aggravated due to FCV-19 in both MHWs and HCWs [17,21,25,32,56,57]. However, we found that FCV-19 explained a significant yet relatively low percentage of STS variation. STS levels in MHWs may be related to pre-existing mental health difficulties aggravated amidst the pandemic, such as personal trauma, depression, anxiety, or sleep problems, which have previously been linked to the severity of STS and PTSD in both MHWs and HCWs [43,53,54]. Furthermore, STS may be related to workplace factors, such as lack of organizational support, workplace violence, rapid and abrupt changes in care protocols without adequate preparation and information [21,52]. All these factors were not explored in this study, as its primary objective was to determine the specific impact of FCV-19 on ProQOL. Female gender increases the vulnerability towards traumatic effect, as was found in the present and previous studies on ProQOL in HCWs or MHWs [32,36,56]. Female gender of HCWs has been associated with a higher risk of poor mental health outcomes, which may be attributed to the increased burden of domestic care, including child and family care that was recorded for women during the pandemic and was combined with excessive workloads [10,58]. Furthermore, it has been suggested that women have a more sensitive hypothalamus–pituitary axis than males, making them more susceptible to stress and fear [59].

Findings about CS in HCWs during the pandemic have been contradictory; some studies found decreased CS [32,56] and others increased [36,60,61]. In our study, MHWs demonstrated moderate levels of CS and no significant association of CS with FCV-19 emerged, suggesting that their content and self-fulfilment in the working environment was not influenced by FCV-19. This finding is in line with prior reports in MHWs [38,39] and could be attributed to the greater coping and emotional regulation strategies that MHWs have been reported to exhibit compared to the general population or other health professionals [62,63]. MHWs’ adaptive and coping capacities based on their professional training and their personal psychotherapeutic resources [64] might have prevented the negative effect of FCV-19 on CS. Resilience has been indicated as a significant predictor of CS in HCWs [61], while training compassion has been found to enhance resilience [65]. Although participants’ resilience levels were not investigated in the current study, previous findings have indicated that MHWs exhibited moderate to high levels of resilience during the COVID-19 pandemic [17]. Furthermore, in our study, older age appears to enhance the ability of MHWs to derive satisfaction from their work and to mitigate negative feelings, potentially due to better psychological adjustment to distressful situations than their younger counterparts, and this may stem from longer professional and personal experience in dealing with adversities. Although we did not demonstrate important differences with regard to years of experience, in recent review studies, less work experience or fewer years of work have been linked to worse mental health outcomes in HCWs during the pandemic [10]. Notably, in the current study, older age was correlated with positive outcomes (greater CS) and younger age with negative (higher BO). The unaffected CS found in MHWs, despite the stressful working conditions during pandemic, may suggest that CS acts as a protective factor against BO and STS, as has been previously evidenced [66].

Our findings have some important public health and policy implications. Severe FCV-19 increases BO and STS, and both have been linked to unfavourable mental health outcomes in MHWs [16,53,54]. Therefore, it is crucial to include in the recommendations that may reduce BO and STS among MHWs, measures that intensify a sense of safety and protection against COVID-19 infections in mental health services [16,17,19,67,68].

### Strengths and Limitations

The results should be considered in light of several strengths and limitations. A significant strength of our study is the estimation of the influence of FCV-19 on ProQOL dimensions, specifically CS, BO, and STS in MHWs, independently and beyond other factors. In addition, it imprints the real-time working experiences of MHWs during the third wave of the pandemic. However, our sample consists of an unbalanced proportion of participants in terms of profession and male–female ratio. Regarding profession, the reason is that MHWs operating remotely were excluded from the study, and as a consequence, occupational therapists were favoured numerically since their work is mostly conducted in person. Regarding gender, the over-representation of females is common in the field of psychology, occupational therapy, and nursing [69,70,71]. Notably, our sample (21% males vs. 79% females) is in concordance with other similar studies [17,21,25]. Finally, we did not examine pre-existing mental health conditions of the participants, and there were no available data regarding pre-pandemic ProQOL of MHWs from different settings in Greece to allow for a comparative interpretation of the results. In addition, the online format of our survey prevented us from measuring the response rates of the targeted respondents. This raises the possibility of self-selection bias since nonrespondents may have been disinterested or too pressured to engage, which may have limited the generalizability of our findings.

## 5. Conclusions

The COVID-19 pandemic represents a new working challenge for MHWs. The negative impact of COVID-19 on the mental wellbeing of MHWs has been broadly ascertained. Our findings indicate that FCV-19 exerts an additional negative influence on MHWs’ mental health and identify groups (younger and female professionals) that are at greater risk for worse ProQOL and mental health outcomes, i.e., burnout and secondary traumatic stress. Organizational support in undertaking actions for the physical and psychological quality of life of MHWs are of prime importance for the quality of care they provide, especially during prolonged crisis situations, such as the COVID-19 pandemic. Interventions that reduce FCV-19, such as specialized psychoeducational programmes [72]; preventing excessive workloads; developing explicit and up-to-date standards and protocols; providing adequate protective equipment and training on protective measures; offering psychological support and free clinical supervision sessions that strengthen resilience; and facilitating social support are essential in preventing BO and STS and in reducing the risk of adverse mental health outcomes [16,17,19,67,68]. At the same time, there is a need for further research in the future on the fear-induced psychological adversities during the pandemic in MHWs with repeated cross-sectional or longitudinal design. Finally, to broaden the understanding of the relationship between fear of COVID-19 and professional quality of life among MHWs and depending on future COVID-19 pandemic conditions, the research team of the current report intends to conduct future research, including professionals working both in the workplace and remotely to enrich the sample and, thus, the generalizability of the study.

## Figures and Tables

**Table 1:** Demographic, professional, and psychological characteristics of the sample.

Characteristics	*n* (%)	Mean (SD)
**FCV-19**		13.76 (4.68)
Extreme FCV-19	53 (23.7%)	
Non-Extreme FCV-19	171 (76.3%)	
**ProQOL**		
Compassion Satisfaction		39.17 (6.19)
Secondary Traumatic Stress		21.48 (6.06)
Burnout Scale		24.28 (6.03)
**Age**		38.27 (10.28)
**Gender**		
Male	47 (21.0%)	
Female	177 (79.0%)	
**Family Status**		
Married	120 (53.6%)	
Single	96 (42.6%)	
Divorced	8 (3.6%)	
**Residence**		
Athens	124 (55.4%)	
Another region	100 (44.6%)	
**Educational level**		
Technical High school	21 (9.4%)	
Graduate degree	123 (54.9%)	
Postgraduate degree (Master/Doctorate)	80 (35.7%)	
**MHWs specialty**		
Occupational therapist	127 (56.7%)	
Nurse	33 (14.7%)	
Psychologist	27 (12.1%)	
Psychiatrist	10 (4.5%)	
Social worker	13 (5.8%)	
Counsellors	14 (6.2%)	
**Service setting**		
Child mental health centre	49 (21.9%)	
Adult mental health centre	15 (6.7%)	
Day hospital	21 (9.4%)	
Day centre	25 (11.2%)	
Psychiatric residential units	58 (25.9%)	
Psychogeriatric hostels	33 (14.7%)	
Psychiatric hospital	6 (2.7%)	
Psychiatric department of a general hospital	8 (3.6%)	
Liaison psychiatric service	9 (3.9%)	
**Monthly income**		
<700 €	20 (8.9%)	
700–1500 €	171 (76.3%)	
1500–2000 €	22 (9.8%)	
>2000 €	11 (4.9%)	
**Professional experience (years)**		10.85 (8.92)

FCV-19: Fear of COVID-19; ProQOL: professional quality of life; MHWs: mental health workers.

**Table 2:** ProQOL dimensions in MHWs with extreme (*n* = 53) and non-extreme FCV-19 (*n* = 171).

	Extreme FCV-19	Non-Extreme FCV-19		
**ProQOL**	**Mean (SD)**	**Mean (SD)**	**Mann–Whitney U**	***p***
Compassion Satisfaction	38.23 (6.5)	39.46 (6.1)	3988.00	0.187
Secondary Traumatic Stress	24.32 (5.9)	20.60 (5.8)	2822.50	<0.001
Burnout	26.80 (5.4)	23.50 (6.0)	3042.50	<0.001

ProQOL: professional quality of life; MHWs: mental health workers; FCV-19: fear of COVID-19.

**Table 3:** The effect of extreme FCV-19 on ProQOL dimensions in MHWs controlling for sociodemographic and professional factors. Hierarchical linear regressions.

Variable	R^2^	F	Model *p*	Beta	t	95.0% Confidence Interval for B		*p*
						Lower Bound	Upper Bound	
Dependent: ***Compassion Satisfaction***								
***Step 1***	0.081	2.67	**0.012**					
Age				0.31	2.47	0.04	0.33	**0.014**
Gender				−0.10	−1.38	−3.48	0.62	0.170
Marital Status				0.03	0.33	−1.44	2.01	0.745
Job				0.04	0.62	−0.51	0.96	0.539
Monthly Salary				0.13	1.86	−0.08	2.75	0.064
Years of Experience				−0.21	−1.84	−0.30	0.01	0.067
Education Level				0.01	0.18	−2.78	3.34	0.857
***Step 2***	0.124	12.46	**0.001**					
Age				0.30	2.43	0.03	0.33	**0.016**
Gender				−0.10	−1.38	−3.48	0.61	0.169
Marital Status				0.03	0.34	−1.42	2.02	0.733
Job				0.04	0.60	−0.51	0.96	0.550
Monthly Salary				0.13	1.82	−0.11	2.72	0.071
Years of Experience				−0.21	−1.82	−0.30	0.01	0.071
Education Level				0.01	0.09	−2.92	3.21	0.926
Extreme fear of COVID-19				−0.08	−1.21	−3.05	0.73	0.229
Dependent: ***Burnout***								
***Step 1***	0.073	2.38	**0.023**					
Age				−0.33	−2.70	−0.34	−0.05	**0.007**
Gender				0.02	0.33	−1.67	2.34	0.742
Marital Status				−0.04	−0.49	−2.12	1.27	0.622
Job				0.06	0.85	−0.41	1.03	0.396
Monthly Salary				−0.06	−0.77	−1.93	0.85	0.443
Years of Experience				0.18	1.57	−0.03	0.27	0.119
Education Level				0.03	0.39	−2.41	3.60	0.697
***Step 2***	0.124	12.46	**0.001**					
Age				−0.32	−2.65	−0.33	−0.05	**0.009**
Gender				0.02	0.35	−1.61	2.30	0.727
Marital Status				−0.04	−0.55	−2.11	1.19	0.582
Job				0.06	0.92	−0.37	1.03	0.357
Monthly Salary				−0.05	−0.67	−1.81	0.90	0.507
Years of Experience				0.17	1.54	−0.03	0.26	0.126
Education Level				0.04	0.65	−1.96	3.91	0.514
Extreme fear of COVID-19				0.23	3.53	1.43	5.05	**0.001**
Dependent: ***Secondary Traumatic Stress***								
***Step 1***	0.073	2.39	**0.023**					
Age				−0.23	−1.89	−0.28	0.01	0.061
Gender				0.18	2.63	0.67	4.70	**0.009**
Marital Status				−0.01	−0.16	−1.83	1.56	0.875
Job				0.03	0.45	−0.56	0.89	0.651
Monthly Salary				−0.01	−0.14	−1.49	1.29	0.889
Years of Experience				0.09	0.74	−0.10	0.21	0.458
Education Level				−0.02	−0.33	−3.52	2.51	0.741
***Step 2***	0.135	15.19	**<0.001**					
Age				−0.22	−1.81	−0.27	0.01	0.072
Gender				0.18	2.73	0.75	4.65	**0.007**
Marital Status				−0.02	−0.21	−1.82	1.47	0.832
Job				0.03	0.52	−0.51	0.89	0.601
Monthly Salary				0.000	−0.01	−1.35	1.34	0.995
Years of Experience				0.08	0.69	−0.10	0.20	0.492
Education Level				−0.00	−0.06	−3.01	2.84	0.953
Extreme fear of COVID-19				0.25	3.90	1.76	5.37	**<0.001**

FCV-19: fear of COVID-19; ProQOL: professional quality of life; MHWs: mental health workers; Statistically significant values are emphasised in bold.

## Data Availability

The data will be made available on request from the corresponding author.
